# Assessment of health service delivery parameters in Kano and Zamfara States, Nigeria

**DOI:** 10.1186/s12913-020-05722-4

**Published:** 2020-09-15

**Authors:** Usaini Bala, Olufemi Ajumobi, Amina Umar, Adefisoye Adewole, Ndadilnasiya Waziri, Saheed Gidado, Audu B. Mohammed, Perpetua Uhomoibhi, Basheer Muhammad, Munira Ismail, Stephen Patrick Kachur, Shelby Cash, Kwame Asamoa

**Affiliations:** 1 African Field Epidemiology Network Nigeria Country Office, Abuja, Nigeria; 2 National Malaria Elimination Programme, Federal Ministry of Health, Abuja, Nigeria; 3 School of Community Health Sciences, University of Nevada, Reno, USA; 4 Kano State Ministry of Health, State Malaria Elimination Programme, Kano, Nigeria; 5 Zamfara State Ministry of Health, State Malaria Elimination Programme, Gusau, Nigeria; 6 Malaria Branch, Division of Parasitic Diseases and Malaria, Center for Global Health, US Centers for Disease Control and Prevention, 1600 Clifton Road, MS- H24-3, 30329 Atlanta, Georgia; 7 Mailman School of Public Health, Columbia University, New York, NY USA

**Keywords:** Data reporting, DHIS2, Malaria surveillance, Master health facility list, Nigeria

## Abstract

**Background:**

In 2013, the Nigeria Federal Ministry of Health established a Master Health Facility List (MHFL) as recommended by WHO. Since then, some health facilities (HFs) have ceased functioning and new facilities were established. We updated the MHFL and assessed service delivery parameters in the Malaria Frontline Project implementing areas in Kano and Zamfara States.

**Methods:**

We assessed all HFs in each of the 34 project local government areas (LGAs) between July and September 2017. Project staff administered a semi-structured questionnaire developed for this assessment to heads of HFs about the type of facility, category and number of staff working at the facility and to record geo-coordinates of facility.

**Results:**

In the Kano State project area, 726 HFs were identified and geo-located: 31 were new facilities, 608 (84%), 116 (16%) and two (0.3%) were Primary Health Care (PHC), secondary and tertiary facilities respectively. Using the national definition, there were 710 (98%) functional facilities and 644 (91%) of these reported to the national health information platform, District Health Information System, version 2 (DHIS2).

The Zamfara project area had 739 HFs: eight were new, 715 (97%), 22 (3.0%) and two (0.2%) PHCs, secondary and tertiary facilities respectively. There were 695 (94%) functional facilities with 656 (94%) of these reporting to DHIS2. Using national criteria for primary health care designation, only 95 (9%) of all PHCs in the two States met the minimum human resource requirements.

**Conclusion:**

Most HFs were functional and reported to DHIS2. A comprehensive MHFL having all the important parameters that should be established and updated regularly by authorities to make it more useful for health services administration and management. Most functional facilities are understaffed.

## Background

Managing health systems depends on having information on the supply and quality of health services available. As countries scale up efforts for improved health coverage and response against major diseases, good data are needed to track the progress and performance of the health system. However, few developing countries have up-to-date data to assess and monitor service quality or to conduct annual review of health services in the public and private sector for the population [1]. Public health decision-making depends greatly on timely availability of good data [2].

The World Health Organization (WHO) encourages countries to develop a comprehensive Master Health Facility List (MHFL) as an initial step towards strengthening performance monitoring at the facility level, which feeds into regional, national, and international monitoring systems [1]. A MHFL is a complete listing of all public and private health facilities in an administrative area. A MHFL should include information about administration and health services provided at each facility [1]. Many countries have several health facility lists with non-standardized norms for identifying and updating the facilities. A MHFL should be standardized with an established unique identifier for each facility, and be linked to other data sources such as the health management information system (HMIS) and logistics management information system (LMIS) to allow information to be compared across time and across data sources.

For many years, Nigeria had different non-standardized health facility lists created for various purposes and development projects. With the growing adoption of information technology in routine health information management, the Nigerian Federal Ministry of Health (FMOH) initiated efforts in 2010 to compile an updated and standardized MHFL. This effort produced a harmonized list in 2013. After the compilation of the list, facilities were assigned unique identifiers, using codes that conveyed information for the states and local government areas (LGAs), but the parameters did not include geo-coordinates [3–5]. In 2017, the FMOH established the national health facility registry (HFR) to update the MHFL via an online platform. The HFR serves as a hub for connecting different information systems, eliminating duplication of health facility lists to enable authorities plan for the establishment of new health facilities [6].

The MHFL does not have a code for the lowest administrative level, the Ward, in its unique identifier [6].. Some facilities have been renamed, relocated, or upgraded, but because the MHFL has not been updated since its creation in 2013, these changes are not reflected in the MHFL. New health facilities constructed after the establishment of the MHFL are also not on the District Health Information Systems version 2 platform (DHIS2), a web-based version of the National Health Management Information System (NHMIS).

In 2016, the US Centers for Disease Control and Prevention (CDC), in collaboration with the Nigeria National Malaria Elimination Program (NMEP), established a 3-year intervention project, Malaria Frontline Project (MFP), with the objectives of strengthening the technical capacity of LGA-level health workers, improving malaria surveillance and facilitating evidence-based decision-making. The project was implemented in Kano and Zamfara States in the Northwest geopolitical zone of Nigeria. During the project implementation, some deficiencies in the list of facilities in DHIS2 and MHFL became obvious. New facilities were submitting monthly data but their data could not be entered into DHIS2 because the facility was not on the list of facilities in DHIS2. Facilities not on the MHFL do not appear on the DHIS2 platform. It became apparent that the analyses and reporting from DHIS2 for the project states did not capture all functional health facilities and non-functional facilities were not identified.

To overcome this problem and improve data reporting and analyses in support of health system surveillance and management at the LGA level, the MFP team together with the State Ministry of Health (MoH) updated the existing MHFL in the project areas in Kano and Zamfara States. In 2017, MFP staff conducted this health facility assessment and submitted the results to the State MoH.

## Methods

### Assessment area

The federal governance system of Nigeria has three tiers of government. The federal level formulates policies, gives implementation guidelines, and sets standards of practice for tertiary care facilities. Nigeria has 36 states and the Federal Capital Territory. The state governments are responsible for translating and implementing federal policies within their states and at the lower levels. The LGA is the next lower tier of governance. Each LGA has 10–15 smaller administrative divisions called Wards. The Ward is a geographical area with a population between 10,000 and 30,000 people and is represented by an elected councilor. Wards are close to the community and are the operational level in the administrative and political system in Nigeria [7–10].

This assessment was conducted in the 20 MFP-implementing LGAs in Kano State (Kano State has a total of 44 LGAs) and in all 14 LGAs in Zamfara State. Kano State has a population of 13,076,892 and Zamfara State has a population of 4,515,427 [11]. Since 2011, all States in Nigeria are using the DHIS2 for monthly health data reporting.

### Assessment design and sampling

The list of facilities on the DHIS2 platform for each LGA was downloaded for the LGA team. All existing facilities, private and public, in each LGA irrespective of their existence in the 2013 MHFL were eligible for the assessment. The LGA team visited each of the facilities from the DHIS2 list in their LGA, and after each interview the person interviewed and other staff at the facility were asked of other facilities in the catchment area. The facility name was cross-checked with the DHIS2 list. If the facility did not appear in the list, the name and address were written down and the team arranged a visit to the new facility.

The assessment used operational definitions from the National Primary Health Care Development Agency (NPHCDA) for minimum standards of Primary Health Care in Nigeria (Table 1) [11]. The category of human resources and the number available at each public PHCs was collected. All non-functional facilities in the assessment area were completely closed and not rendering any health services.

**Table 1:** Operational definitions of minimum standards for Primary Health Care in Nigeria

Functional health facility: A place where clinical services are provided by health care workers, utilized by clients, and has a suitable infrastructure. All functional health facilities are to submit monthly reports of aggregated data into NHMIS using the monthly summary form.	
**Primary Health Care (PHC)**	consists of Health Post, Health Clinic and Primary Health Centre
The minimum human resource requirements for PHC	
**Health Post**	Junior Community Health Extension Worker (JCHEW) – 1, Health attendant - 1
**Health Clinic**	Nurses/Midwife – 2, Community Health Extension Worker (CHEW) – 2, JCHEWs - 4, Health attendant/assistant - 2
**Primary Health Centre**	Medical doctor - 1, Community Health Officer (CHO) - 1, nurses/midwife - 4, Pharmacy technician - 1, CHEW - 3, JCHEW - 6, Environmental Health Officer (EHO) - 1, Medical Records Officer - 1, Laboratory technician - 1, Health attendant/assistant - 2

### Data collection

MFP staff working at the LGA level are known as Malaria National Stop Transmission of Polio Local Government Officers (MNSLOs). The MNSLOs live in the LGAs where they work and share office with the LGA malaria team. At the time of the survey, the MNSLOs had been on post a little over 1 year. The project team at national level developed the structured questionnaire which was pretested. For pretesting, the field team administered the questionnaire to health care workers in a non-project area and feedback was incorporated into the final questionnaire. The MNSLOs were trained to administer the questionnaire and collect data using open data kit (ODK) on android-based phones [12]. ODK Collect is an open source (free) Android app that replaces paper forms used in a survey-based data gathering. Data were collected from July to September 2017. The MNSLOs collected the data during their routine health facility supportive supervision visit. The MNSLOs identified health facilities using the health facility list from the DHIS2 platform. Health facility heads or their assignee in functional facilities were interviewed. A non-functional facility (facility that was completely closed), was confirmed by asking the owner of the closest residence. This was done since there was no additional data source or official communication on facility closures. At the end of each interview the MNSLO recorded the geo-coordinates of the facility using the GPS component of the phone. Data were collected on the functional status of the facility, the number of personnel in each staff category working at the facility, type of facility, ownership of facility, type of register being used by facility, current or old version and whether the facility currently submits reports to DHIS2. At the time of the assessment, some facilities were still using the old version of register which did not collect all indicators required by DHIS2.

### Data processing and analysis

Data collected on ODK were transmitted daily to a server. The data were downloaded at the end of the study and analyzed using Microsoft Excel version 16. The frequencies and proportions were calculated.

## Results

### Health facilities

**Fig. 1:**
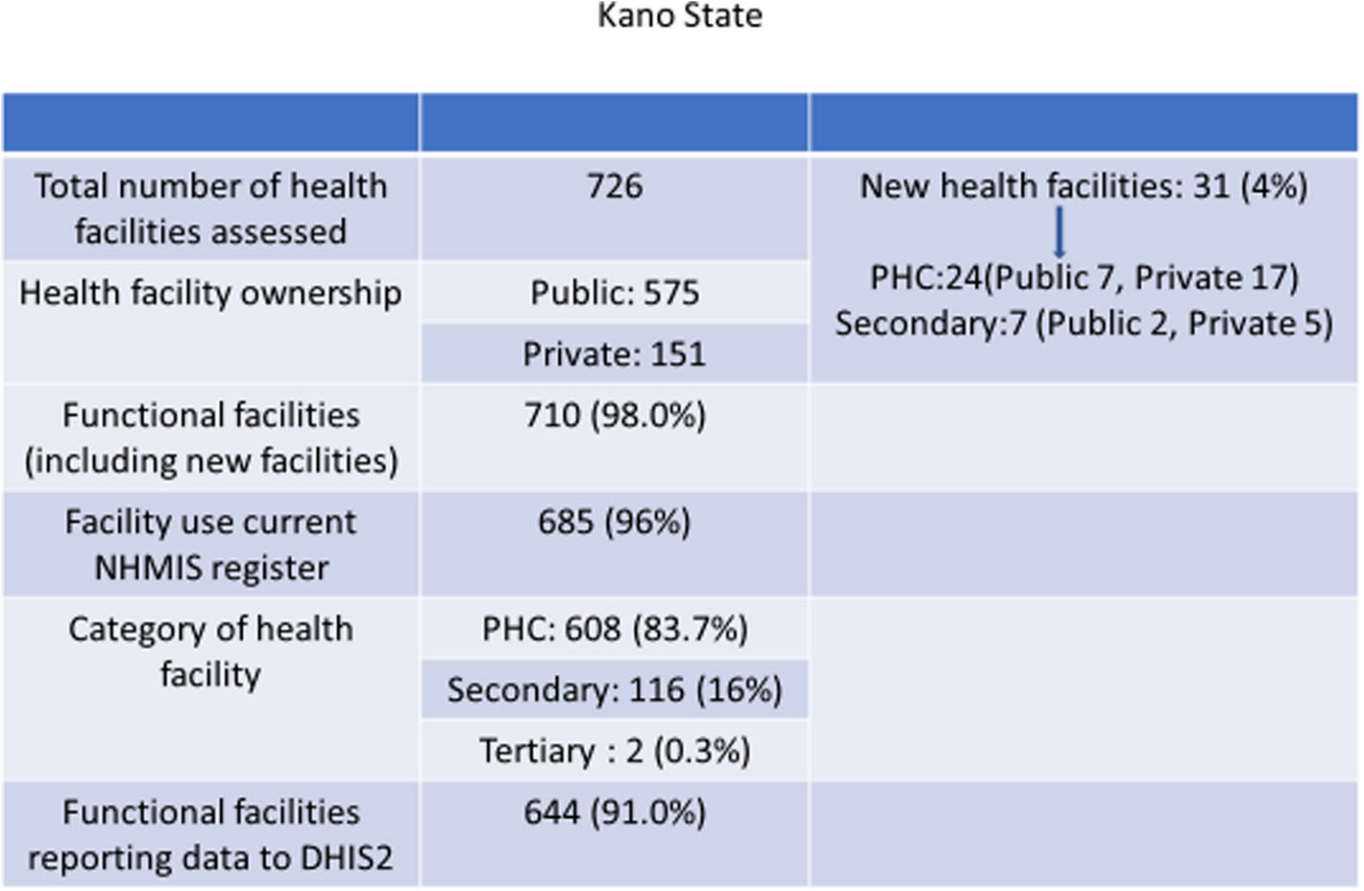
Chart of health facilities assessed in Kano States showing category, ownership, functionality, use of HMIS and reporting to DHIS2

**Fig. 2:**
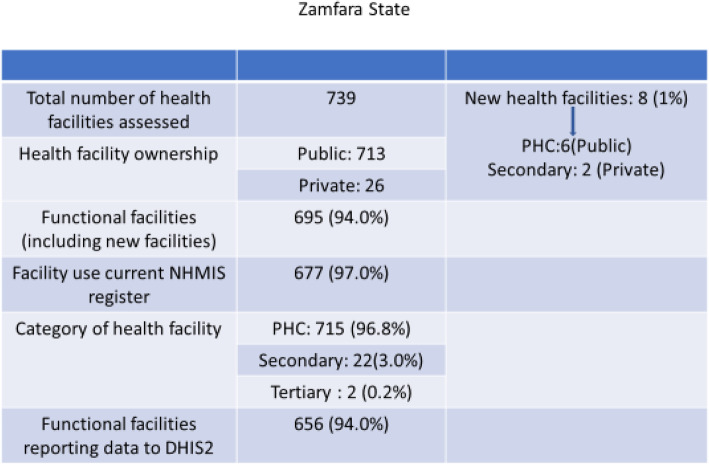
Chart of health facilities assessed in Zamfara States showing category, ownership, functionality, use of HMIS and reporting to DHIS2

In the Kano State MFP implementing LGAs, 726 health facilities were found, of which 31 (4%) were new facilities. All new facilities were not on the DHIS2 platform. Of the 726 health facilities assessed, 575 (79%) were public facilities. Sixteen non-functional facilities were identified, made up of 12 PHCs and four secondary facilities (Fig. 1). There were 435 PHCs of which 276 (63%) were health posts. In Zamfara State, 739 health facilities were found, eight (1%) were new facilities. Six of the new public facilities were PHCs and two were secondary-level facilities (Fig. 2). There were 43 (98%) public PHCs among the 44 non-functional facilities. Of the 639 functional public PHCs, 396 (62%) were Health Posts.

### Distribution of health facilities and unique identification numbers

**Fig. 3:**
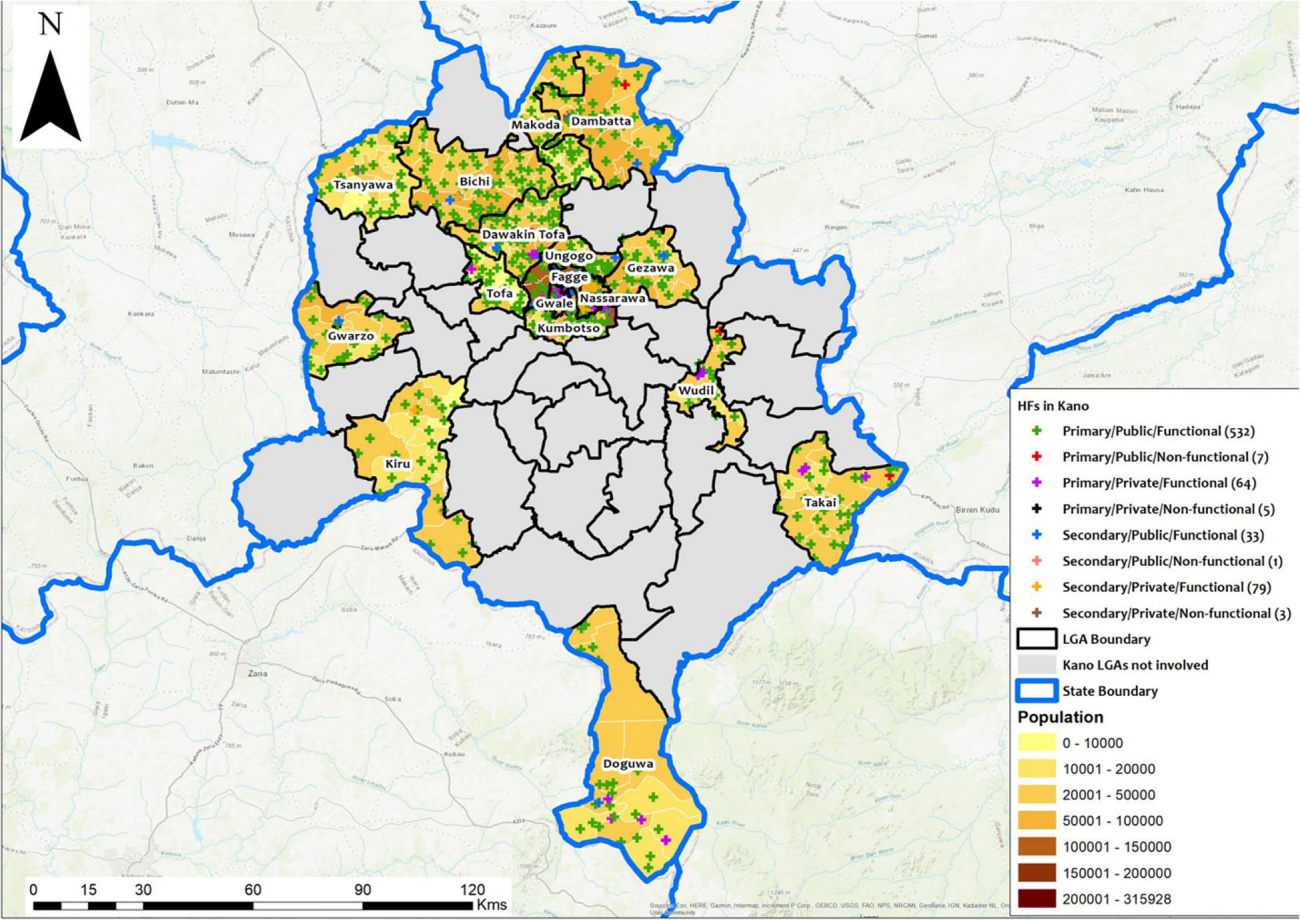
Map of Kano State showing project LGAs

**Fig. 4:**
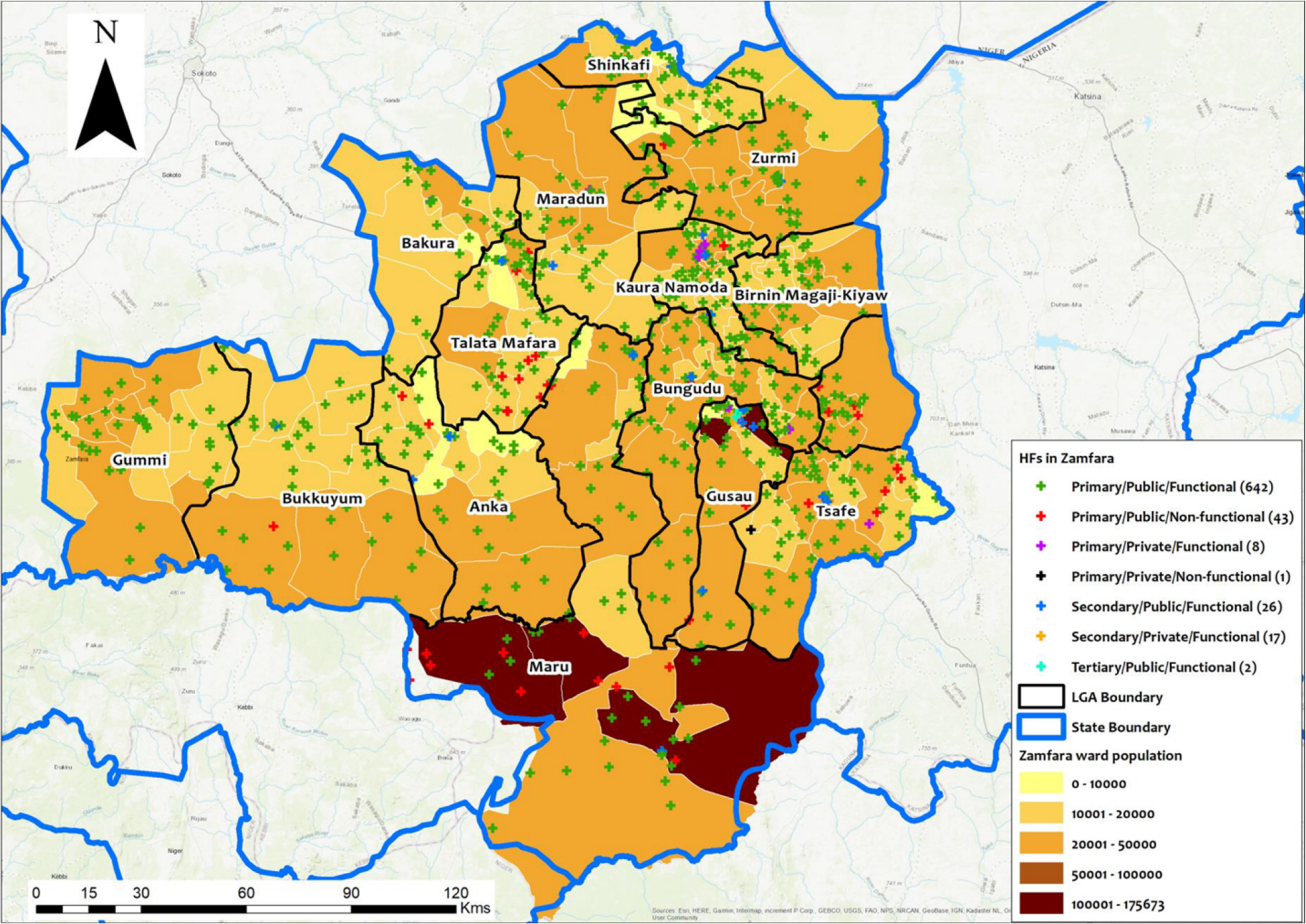
Map of Zamfara State showing project LGAs

All health facilities assessed had their geo-coordinates recorded for use in geo-mapping. As observed in the Kano State map, the PHC distribution in Doguwa LGA show more facilities concentrated in the southern area while the middle and northern areas of the LGA with higher population density have fewer facilities (Fig. 3). The spatial distribution of facilities in the LGAs of Zamfara State show less health facilities in the high population density areas in the southern part of the state (Fig. 4).

### Human resource at public PHCs

Human resources (HR) of public PHCs (excluding PHCs owned by a tertiary institution, police and prisons services, secondary, tertiary, and private health facilities) was compared with the NPHCDA HR minimum guidelines. The NPHCDA guidelines state the categories of health personnel and the minimum number to deliver the requisite services in each class of facility. A summary of category and number of health personnel working at the various public PHCs in the project area are presented in Table 2.

**Table 2:** Category and number of health workers at government Primary Health Care Centers in Zamfara and Kano States

Designation	Kano State									Zamfara State								
	Health Post ***N*** = 276 n (%)			Health Clinic ***N*** = 43 n (%)			Primary Health Centre ***N*** = 116 n (%)			Health Post ***N*** = 396 n (%)			Health Clinic ***N*** = 90 n (%)			Primary Health Centre ***N*** = 153 n (%)		
	Actual	Expected	Gap	Actual	Expected	Gap	Actual	Expected	Gap	Actual	Expected	Gap	Actual	Expected	Gap	Actual	Expected	Gap
**Doctors**	0 (0.0)	–	–	4 (0.5)	–		18 (1.3)	116	98	0 (0.0)	–	–	0 (0.0)	–		1 (0.1)	153	152
**Nurses**	1 (0.1)	–	–	23 (2.9)	86	39	28 (2.0)	464	355	2 (0.2)	–	–	4 (0.9)	180	175	7 (0.5)	612	536
**Midwife**	9 (1.3)	–	–	24 (3.1)			81 (5.8)			6 (0.7)	–	–	1 (0.2)			69 (4.6)		
**Pharmacist**	8 (1.2)	–	–	3 (0.4)	–	–	9 (0.6)	–	–	0 (0.0)	–	–	0 (0.0)	–	–	1 (0.1)	–	–
**Lab. Scientist**	0 (0.0)	–	–	3 (0.4)	–	–	14 (1.0)	–	–	0 (0.0)	–	–	0 (0.0)	–	–	0 (0.0)	–	–
**CHO**	5 (0.7)	–	–	13 (1.7)	–	–	32 (2.3)	116	84	4 (0.4)	–	–	5 (1.1)	–	–	32 (2.2)	153	121
**CHEW**	151 (22.0)	–	–	210 (27.0)	86	+ 124	344 (24.5)	348	4	244 (27.2)	–	–	107 (23.1)	180	73	257 (17.4)	459	202
**JCHEW**	166 (24.2)	276	110	115 (14.8)	172	57	187 (13.3)	696	509	165 (18.4)	396	231	56 (12.1)	360	304	138 (9.3)	918	780
**Lab. Technicians**	18 (2.6)	–	–	35 (4.5)	–	–	107 (7.6)	116	9	34 (3.8)	–	–	25 (5.4)	–	–	156 (10.6)	153	
**Pharmacy Technicians**	4 (0.6)	–	–	19 (2.4)	–	–	46 (3.3)	116	70	7 (0.8)	–	–	12 (2.6)	–	–	40 (2.7)	153	113
**Record officer**	16 (2.3)	–	–	30 (3.8)	–	–	60 (4.3)	116	56	34 (3.8)	–	–	31 (6.7)	–	–	102 (6.9)	153	51
**Health attendant**	105 (15.3)	276	171	113 (14.5)	86		161 (11.5)	232	71	143 (15.9)	396	253	95 (20.5)	180	85	221 (15.0)	306	85
**Health assistant**	32 (4.7)	–	–	35 (4.5)	–	–	84 (6.0)	232	148	127 (14.1)	396	269	69 (14.9)	180	111	254 (17.2)	–	–
**Environmental Health Officer**	145 (21.1)	–	–	133 (17.1)	–	–	179 (12.7)	116		128 (14.3)	–	–	44 (9.5)	–	–	158 (10.7)	153	
**Lab. Assistant**	11 (1.6)	–	–	12 (1.5)	–	–	46 (3.3)	–	–	3 (0.3)	–	–	11 (2.4)	–	–	29 (2.0)	–	–
**Lab. Attendant**	8 (1.2)	–	–	2 (0.3)	–	–	5 (0.4)	–	–	1 (0.1)	–	–	3 (0.6)	–	–	7 (0.5)	–	–
**Pharmacy assistant**	8 (1.2)	–	–	4 (0.5)	–	–	5 (0.4)	–	–	0 (0.0)	–	–	0 (0.0)	–	–	5 (0.3)	–	–
**Total**	687 (100.0)			778 (100.0)						898 (100.0)			463 (100.0)			1477 (100.0)		

In Kano State, 317 (46%) of staff in health posts were CHEWs/ JCHEWs and 137 (20%) were Health assistants/ attendants. In health clinics, CHEW/ JCHEW were 325 (42%) and Health assistants/ attendants made up 148 (19%). There were excess CHEW at the Health Clinics, about 144%. Among Primary Health Centres, 531 (38%) of the staff were CHEW/ JCHEW and 245 (18%) were Health assistants/ attendants (Table 2). Of the 276 health posts, 56 (20%) met the minimum HR requirement. None (0%) of the 43 functional health clinics met the minimum HR requirement only one (0.9%) of the 116 PHCs met the minimum HR requirement.

At health posts in Zamfara State, 409 (46%) of the staff were CHEW/ JCHEW and 270 (30%) were health assistants/attendants. Among health clinics, 163 (35%) were CHEW/ JCHEW and 164 (35%) were health assistants/ attendants. At Primary Health Centres, 395 (27%) were CHEW/ JCHEW and 475 (32%) were health assistants/ attendants (Table 1). Of the 398 health posts in Zamfara State, 36 (9%) met the minimum HR requirement. One (1%) of 87 health clinics and one (0.6%) of 154 PHCs met the minimum human resource requirement.

### Facility data reporting to district health information system

In Kano, there were 710 functional health facilities and 685 (96%) used the recommended NHMIS registers. Six hundred and forty-four facilities (91%) submitted monthly reports to LGA M&E officer for entry into the DHIS2 platform (Fig. 1). In Zamfara, there were 695 functional health facilities and 677 facilities (97%) used the required NHMIS registers. Six hundred and fifty-six facilities (94%) submitted monthly reports to LGA M&E officer for entry into the DHIS2 platform (Fig. 2). Anecdotal reports from NSLOs indicate that some health workers did not submit reports because they did not understand how to complete the forms. Others had not been trained or did not have the current registers.

## Discussion

This assessment of the parameters recorded for MHFL and the staff cadre and number available at facilities shows the importance of having up to date MHFL, which will provide comprehensive data that will be comparable over time and useful for administrative and managerial purposes. A comprehensive geocoding and identification codes captured during the assessment offers all information that identifies each health facility to facilitate administrative support and show the type of health services expected from the facility. MHFL is crucial for disease surveillance and health system management [1]. In the 4 years since the MHFL was compiled, the number of functional health facilities increased by 4.3 and 1.1% in Kano and Zamfara States, respectively, while at the same time 2.2 and 6.0% of listed health facilities were non-functional in Kano and Zamfara States, respectively. The data collection for this assessment added no extra cost to the project. MNSLOs regularly visit facilities to support the program implementation and collected the assessment data during the visit.

The establishment of the HFR is important to serving as the responsible office to coordinate and collect all useful public comments from stakeholders. The HFR is meant to build on MHFL of 2013 and decide on parameters to include in the MHFL as well as the frequency of updating the MHFL. The addition of facilities to the MHFL is at the recommendation of the HFR.

This assessment has provided the Ministry of Health an updated MHFL with geo-coordinates for the two states. The geo-coordinates of the facilities are helpful for future planning and decision-making by health authorities on setting priority facilities or identifying deprived areas to locate new facilities or which facility to upgrade for effective service delivery beneficial to the population. Distance influences access and use of health services. In case of disease outbreaks or unexpected health outcomes identified in NHMIS or DHIS2, authorities will be able to better plan and intervene with the necessary control measures knowing the geographical distribution of facilities [13, 14]. It will be beneficial to establish Ward level unique identification number for facilities.

The MHFL should be updated and linked to the human resources database of each health facility. The link between the MHFL and the HR database will help health managers to determine staff needs and postings on an evidence base. Facilities may be understaffed by not having adequate numbers or by lacking certain cadres of personnel. Understaffing negatively influences quality of services provided by facilities [15, 16]. Having 20% of health posts in Kano State project area and less than 10% of health posts in Zamfara State meeting the minimum HR requirements is an issue worth health authorities’ attention. In all categories of PHC facilities, most staff are CHEW and JCHEW; CHOs are in few facilities. In Zamfara State, only one doctor was posted at a Primary Health Centre. Seven percent of the Primary Health Centres in Kano State have doctors, but these facilities function as Comprehensive Health Centers, similar to findings in Edo State by Alenoghena I.O et al. [17]. The excess CHEW staff at Health Clinic in Kano State should be addressed for they might be underutilized. The assessment did not collect information on patient load and quality of services provided. However, gaps in HR requirements observed from the study could be addressed through targeted employment of required health personnel and redistribution of health personnel to areas of their greatest need. The majority of DHIS2 listed health facilities in Kano State (91%) and Zamfara State (94%) report data into DHIS2 platform. This is very important as most facilities provide the needed data to analyze and make informed decisions.

## Conclusions

The assessment helped update the MHFL in the 34 MFP LGAs. The functional status of facilities was documented as well as the geo-coordinates of the health facilities. HR requirements for government public facilities was also assessed. These data will help LGA and state health authorities know the number of facilities expected to report to DHIS2. Though most PHCs are functional and deliver health services to the population, the low number of facilities within PHC meeting the minimum HR requirement will hamper the countries effort to achieve its goal of universal health coverage. PHCs in the project area need more technical staff. The parameters being collected in the present MHFL is not comprehensive enough for health service management. We suggest adding unique identification numbers for Wards and a human resources database for each facility. Such comprehensive database will help authorities to objectively plan for health facilities and posting of health workers to achieve universal health coverage.

## Supplementary information

**Additional file 1.**

**Additional file 2.**

## Data Availability

All data generated or analyzed during this study are included in this published article and if any additional data set is needed, it can be made available by author to the publisher at any time.

## Abbreviations

WHO: World Health Organization
MHFL: Master Health Facility List
HMIS: Health Management Information System
LMIS: Logistics Management Information System
FMOH: Federal Ministry of Health
LGAs: Local Government Areas
HFR: Health Facility Registry
DHIS2: District Health Information Systems version 2 platform
NHMIS: National Health Management Information System
CDC: Centers for Disease Control and Prevention
NMEP: Nigeria National Malaria Elimination Program
MFP: Malaria Frontline Project
MoH: Ministry of Health
NPHCDA: National Primary Health Care Development Agency
MNSLOs: Malaria National Stop Transmission of Polio Local Government Officers
ODK: Open Data Kit
HR: Human resource
PHCs: Primary Health Centres
CHEWs: Community Health Extension Workers
JCHEWs: Junior Community Health Extension Workers
CHOs: Community Health Officers

## References

[1] Creating Master Health Facility List, WHO 2013. [Internet]. Available from: https://www.who.int/healthinfo/systems/WHO_CreatingMFL_draft.pdf?ua=1 (accessed April 13,2019.

[2] Makinde O, Meribole E, Oyediran K, Fadeyibi F, Cunningham M, Hussein-Fajugbagbe Y, Toye F (2018). Duplication of effort across development projects in Nigeria: an example using the master health facility list. Online J Public Health Inform.

[3] Makinde O, Azeez A, Bamidele S, Oyemakinde A, Oyediran K, Adebayo W (2014). Development of a master health facility list in Nigeria. Online J Public Heal Inform.

[4] Makinde O, Azeez A, Adebayo W (2016). Potential use cases for the development of an electronic health facility registry in Nigeria: key informant’s perspectives. Online J Public Heal Inform.

[5] AbouZahr C, Boerma T (2005). Health information systems: the foundations of public health. Bull World Health Organ.

[6] Nigeria Health Facility Registry (HFR).

[7] Using DHIS2 to Strengthen Health systems. [Internet]. Available from: https://www.measureevaluation.org/resources/publications/fs-17-212.

[8] Abosede O, Campbell P, Olufunlayo T, Sholeye O (2012). Establishing a sustainable ward health system in Nigeria: are key implementers well informed?. J Community Med Heal Educ.

[9] Federal Republic of Nigeria (2004). revised National Health Policy.

[10] Uzochukwu B, Ajuba M, Onwujekwe O, Nkoli E (2011). Examining the links between accountability, trust and performance in health service delivery in Orumba south local government area, Nigeria [internet].

[11] National Bureau of Statistics, 2017 Demographic Statistics Bulletin.

[12] Open data kit [Internet]. Available from: https://docs.opendatakit.org/.

[13] Minimum Standards for Primary Health Care in Nigeria. National Primary Health Care Development Agency, Federal Government of Nigeria.

[14] Rose-Wood A, H N, Thermidor R, Chan J, Joseph F, Lerebours G (2014). Development and use of a master health facility list: Haiti’s experience during the 2010 earthquake response. Glob Heal Sci Pr [Internet].

[15] Lankshear A, Sheldon T, Maynard A (2005). Nurse staffing and healthcare outcomes: a systematic review of the international research evidence. Adv Nurs Sci [Internet] [Internet].

[16] Shirom A, Nirel N, Vinokur A (2006). Overload, autonomy, and burnout as predictors of physicians’ quality of care. J Occup Heal Psychol [Internet].

[17] Alenoghena I, Isah E, Isara A (2016). Availability and type of human resource for health in public primary health care facilities in selected communities, Edo state. J Community Med Prim Heal Care.

