# Supplementary figures and images for: Assessment of health service delivery parameters in Kano and Zamfara States, Nigeria
Source: BMC Health Serv Res. 2020 Sep 15;20:874. doi: 10.1186/s12913-020-05722-4 (PMC7493332; doi:10.1186/s12913-020-05722-4)

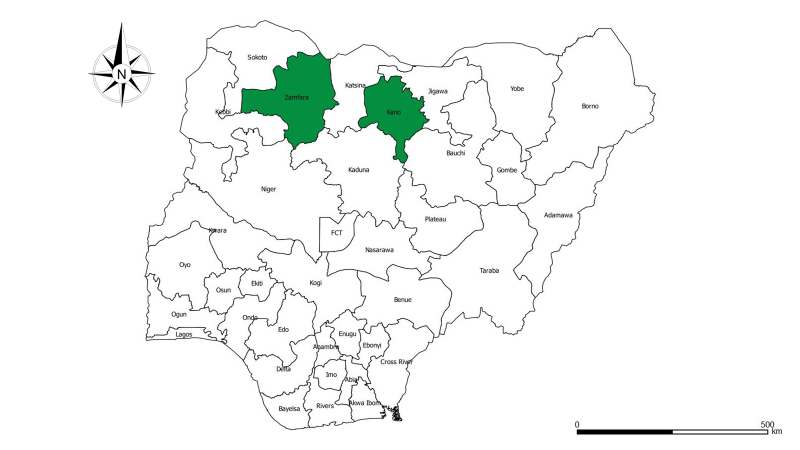

Supplement: Supplementary file 1 — Additional file 1. [file 12913_2020_5722_MOESM1_ESM.docx]
